# Cognition is selectively impaired in males with spinal pain: A retrospective analysis of data from the Longitudinal Study of Ageing Danish Twins

**DOI:** 10.1113/EP091177

**Published:** 2024-02-17

**Authors:** David C. Byfield, Benjamin S. Stacey, Damian M. Bailey

**Affiliations:** ^1^ Neurovascular Research Laboratory, Faculty of Life Sciences and Education University of South Wales Pontypridd UK

**Keywords:** cognitive function, dementia, neuroinflammation, physical inactivity, spinal pain

## Abstract

Cognitive decline and spinal pain (back pain [BP] and neck pain [NP]) represent a major public health challenge, yet the potential relationship between them remains elusive. A retrospective analysis of the Longitudinal Study of Ageing Danish Twins was performed to determine any potential relationships between BP/NP and cognitive function adjusting for age, sex, educational and socioeconomic status. A total of 4731 adults (2788 females/1943 males) aged 78 ± 6 (SD) years were included in the analysis. We observed a 1‐month prevalence of 25% with BP, 21% with NP and 11% for combined BP/NP. While there were no differences in cognition scores for males and females reporting combined BP/NP, compared to those without combined BP/NP (34.38 points [95% confidence interval (CI) = 31.88, 36.88] vs. 35.72 points [95% CI = 35.19, 36.26]; *P* = 0.180; and 35.72 points [95% CI = 35.19, 36.26] vs. 35.85 points [95% CI = 35.39, 36.31]; *P* = 0.327; for male and females, respectively), an adjusted analysis revealed that males with combined BP/NP presented with lower cognitive scores compared to males without combined BP/NP (81.26 points [95% CI = 73.80, 88.72] vs. 79.48 points [95% CI = 70.31, 88.66]; *P* = 0.043). The findings of this hypothesis‐generating study may highlight a potential sex‐specific association between spinal pain and later‐life neurodegeneration.

## Abstract (highlights)

- **What is the central question of this study?**
  Do adults reporting spinal pain (neck pain and/or low back pain) exhibit lower cognitive function compared to pain‐free controls, and to what extent does this relationship differ as a function of biological sex?
- **What is the main finding and its importance?**
  In elderly males reporting spinal pain, cognitive function was selectively lower compared to females following adjustment for age, sex, educational and socioeconomic status. These findings identify spinal pain as a potential modifiable risk factor for latent neurodegeneration.

## 1 INTRODUCTION

Cognitive decline and dementia represent a major health and social care challenge in the 21st century as a leading cause of global morbidity and mortality (Livingston et al., 2020). Current estimates indicate that over 55 million people worldwide are living with some form of dementia and this will further increase to 152 million by 2050, doubling every 5 years (Cao et al., 2020; Livingston et al., 2020; Nichols et al., 2019; Oudbier et al., 2022). In 2019, it was estimated that 1.6 million deaths worldwide were attributable to dementia (Nichols & Vos, 2020), with recent evidence suggesting that this is likely an underestimate (Stokes et al., 2020). Alzheimer's disease, the most common form of dementia, affects females disproportionately more than men particularly in later life, and females currently represent two‐thirds of patients with Alzheimer's disease worldwide (Goldstein et al., 2021; Hebert et al., 2001). While the difference between males and females cannot simply be ascribed to enhanced (female) longevity (Nebel et al., 2018), the precise sex‐specific risk factors and differential mechanisms that underlie vulnerability remain unclear (Goldstein et al., 2021; Hebert et al., 2001; Hendriks et al., 2021; Launer, 2019; Levine et al., 2021; Wolters et al., 2020).

Back pain (BP) and neck pain (NP), often referred to collectively as spinal pain (Ferreira & de Luca, 2017), are also regarded as a serious public health concern, affecting 610 million people globally in 2020 and projected to reach 843 million by 2050 (Ferreira et al., 2023). Acknowledged as the primary cause of years lived with disability worldwide (Hartvigsen et al., 2018), spinal pain often co‐exists with cognitive decline and neurodegeneration (Ferreira & de Luca, 2017; Williams et al., 2018), contributing to reduced functional capacity and lower overall quality of life (Bailey et al., 2018). Older patients with persistent spinal pain are more likely to exhibit cardiovascular disease risks factors (e.g., hypertension, smoking, type 1 and 2 diabetes, anxiety, physical inactivity, depression, cancer, chronic inflammatory disease) (Landau & Harrison, 2021; Liaghat et al., 2023; Rönnegård et al., 2022), in conjunction with increased risk of myocardial infarction, stroke and cardiovascular death independent of these cardiovascular risk factors (Fernandez et al., 2017; Rönnegård et al., 2022). Evidence is also emerging that those presenting with spinal pain may suffer from multimorbidity, defined as the co‐existence of two of more chronic conditions (Willadsen et al., 2016), such as obesity, diabetes, pulmonary disease, cardiovascular disease, osteoporosis, asthma, migraine and osteoarthritis (de Luca et al., 2017; Rafn et al., 2023). Chronic spinal pain appears to be more debilitating in older adults, contributing to decreased mobility, fewer social interactions and a sedentary lifestyle, which may adversely impact cognitive function (Ferreira & de Luca, 2017; Rafn et al., 2023). Of interest, it is also acknowledged that females report a much higher prevalence of both spinal pain and dementia across the globe (Levine et al., 2021; Wu et al., 2020).

Despite their co‐existence, our current understanding of the underlying physiological mechanisms that potentially link spinal pain and accelerated cognitive decline remains unclear (Zhou et al., 2022). A reduction in brain volume (grey matter) and connectivity in those with spinal pain has been suggested (Yu et al., 2021), yet the underlying mechanisms here remain obscure. From a physiological perspective, the molecular and haemodynamic consequences of elevated oxidative–inflammatory–nitrosative stress (OXINOS), either as a cause or consequence of spinal pain and/or compounded by physical inactivity, may prove a unifying physiological mechanism. In support, OXINOS defined by a free radical‐mediated reduction in vascular nitric oxide bioavailability, inflammation, vascular endothelial dysfunction and structural damage to the neurovascular unit, has been shown to precede cognitive decline and later‐life neurodegeneration (Bailey et al., 2019). Moreover, it has been suggested that a close link exists between the somatosensory and nociceptive nervous systems (Khera & Rangasamy, 2021), given that pain and cognition share common neural substrates that are known to interact reciprocally (Moriarty & Finn, 2014; Moriarty et al., 2011).

Although the relationship between pain perception and cognitive function is evident, the moderating role of age and sex in the pain–cognition relationship remains to be established (Oosterman et al., 2013). Therefore, we sought to determine the potential relationship(s) between spinal pain (defined as back pain and neck pain) and cognitive decline, including its modulation by age and sex, which has not been previously investigated. This hypothesis‐generating study constituted an exploratory retrospective analysis of historical datasets from the Longitudinal Study of Ageing Danish Twins (LSADT) database held in the Danish Twins Registry (Skytthe et al., 2013). We reasoned that elderly adults reporting spinal pain would exhibit lower cognition scores, and that this would be more pronounced in females, given their established vulnerability to later‐life neurodegeneration and higher age‐standardised prevalence.

## 2 METHODS

### 2.1 Ethical approval

This study was approved by the Faculty of Life Sciences and Education Ethics Committee at the University of South Wales (no. 19DB0501LR) and Danish Data Protection Agency (2007‐54‐0227). Written informed consent was obtained, and the study conformed to the standards set by the latest revision of the *Declaration of Helsinki*, except for registration in a database (World Medical Association, 2013).

### 2.2 Design and participants

A cross‐sectional analysis of data obtained from the LSADT database, a nationwide population‐based cohort‐sequential study of 70+‐year‐old Danish twins (monozygotic, dizygotic and opposite‐sex dizygotic) born between 1870 and 1930 (Fernandez et al., 2017; Kyvik et al., 1995; Pedersen et al., 2019) was performed. The LSADT is a questionnaire‐based omnibus study and between the years of 1995 and 2001, home‐based interviews were conducted every 2 years by trained interviewers from the Danish National Institute of Social Research providing an opportunity to investigate relationships between a variety of health‐related metrics (Skytthe et al., 2002, 2011, 2013). The interviews lasted 60−75 min and consisted of an extensive battery of questionnaires designed to document socio‐demographics and record overall general health including physical functioning, cognition, associated co‐morbidities and other lifestyle factors (Christiansen et al., 2003; McGue & Christensen, 2007). We specifically examined data collected for both males and females in all four recorded LSADT intake points in 1995, 1997, 1999 and 2001. The 1995 intake was collected for participants aged 75 years and older. In 1997, Danish twin pairs aged 73 years or older were invited to participate and in 1999 and 2001, twins aged 70+ years were included.

### 2.3 Measures

#### 2.3.1 Spinal pain (back and neck pain)

BP was assessed using the question, ‘Have you during the past month suffered from back pain, acute back pain or lumbago?’ and NP was assessed using the question, ‘Have you during the past month suffered from pain or stiffness in the neck or shoulders?’. Response options to both questions were dichotomised as ‘Yes’ or ‘No’. The data were subsequently divided into four groups as those who reported: (1) no pain; (2) BP only; (3) NP only; and (4) combined BP and NP (Hartvigsen et al., 2003, 2004). No additional (retrospective) information regarding the duration (acute or chronic), intensity and frequency of the reported pain was available to complement the current analyses.

### 2.4 Cognitive function

Cognitive function was assessed using a battery of five standardised cognitive tests shown in previous studies to be a valid, reliable, positively correlated and age‐sensitive measure of global cognitive function, moderately stable over a 2 year interval and having a high internal consistency reliability (0.75) as reported in previous studies (Dokkedal et al., 2016; McGue & Christensen, 2001, 2013; Vestergaard et al., 2015). The five specific tasks included: (1) a category fluency task in which participants were asked to name as many animals as they could during a 1‐min interval; (2) a forward digit span test; (3) a backward digit span test; (4) an immediate recall of a 12‐item list; and (5) a delayed recall of a 12‐item list (Osler et al., 2007). An overall composite measure of global cognitive function was calculated by summing the five standardised scores to yield a composite *z*‐score based on the means and standard deviation of the 70+‐year‐old individual twins. The composite scoring ranged from 0 to 80 points with a lower score indicative of poor(er) cognitive performance (Dokkedal et al., 2016). This composite of five individual cognitive measures was chosen to represent tasks that are sensitive to normative age changes that can be reliably and briefly assessed. The inter‐correlations of the cognitive components range from 0.33 to 0.46 (McGue & Christensen, 2001) and the five individual cognitive measures are temporally stable and positively correlated, rationalising the formation of a composite of the five tests (McGue & Christensen, 2007). Standardisation of the scores for all the surveys was based on the component means and standard deviation observed in the 1995 intake permitting direct comparison of the composite scores across the multiple waves of assessment (1995, 1997, 1999, 2001). While these cognitive measures are not sufficiently sensitive to detect a change in cognitive status between time points, they are able to assess verbal ability (fluency), executive control, short term working memory performance, attention span and the integrity of the semantic memory (ability to access and retrieve knowledge), encapsulating a basal assessment of cognitive function for this analysis (Vestergaard et al., 2015).

### 2.5 Educational status

Educational status was also recorded as an ordinal measure based on self‐report in six categories by combining questions related to basic and vocational schooling as reported in previous studies (Dokkedal et al., 2018; Jensen & Rasmussen, 2011). This was categorised as: (1) no vocational education and less than 7 years of education; (2) no vocational education and more than 7 years of education; (3) vocational education and 7–9 years of education; (4) vocational education and more than 9 years of education; (5) short and medium education; and (6) long‐term education.

### 2.6 Socio‐economic status

Socioeconomic class was based on classification from Statistics Denmark (Statistics Denmark‐Socio‐Economic Classification—SOCIO13, v1:2014), as follows: (1) social class I, university graduates; (2) social class II, self‐employed (with 6−20 subordinates) and salaried employees; (3) social class III, self‐employed (5 employees) and specialised work; (4) social class IV, salaried employees, lower level and skilled manual workers; and (5) social class V, unskilled manual workers (Dokkedal et al., 2016, 2018).

### 2.7 Comorbidities

Data regarding the frequency and impact of additional competing cardiopulmonary and/or cerebrovascular comorbidities were not captured during the interview process and therefore not available within this analysis.

### 2.8 Statistical analysis

Inferential analyses based on independent sampling were performed using Stata (version 16.1, StataCorp, College Station, TX, USA) led by the senior biomedical statistician (Lisbeth Aagaard Larsen, see Acknowledgements). Age at intake, sex ratio, and counts for educational and socioeconomic status, and BP and/or NP, and cognitive function were described. The chi‐square test was employed to determine differences in frequency counts of BP and NP between male and females. Repeated Shapiro–Wilk W tests confirmed that all datasets were normally distributed (all *P* > 0.05). Student's independent samples *t*‐test was employed to determine differences in cognitive function between adults reporting NP/BP versus those not reporting NP/BP, first for pooled data and then according to sex. Multivariable linear regression analysis was used to determine potential associations between BP/NP and cognitive function, adjusting for age at intake interview, sex, educational and socioeconomic status. The covariates selected for this study were informed by a prior publication given their established modulatory impact(s) on cognitive function in a comparable demographic (Dokkedal et al., 2016, 2018). Data were presented using means ± standard error and 95% confidence intervals (CI) with significance established for all two‐tailed tests at *P* < 0.05.

## 3 RESULTS

### 3.1 Sample size

Data were obtained from 4731 adult twins (2788 females/1943 males) including 1585 monozygotic, 280 dizygotic, 224 opposite sex dizygotic and 32 unknown zygosity twins with a mean age of the 78 ± 6 years in whom 4164 (88%) completed the standardised cognitive tests. Age at intake, educational status and socio‐economic status are summarised in Table 1.

**TABLE 1:** Participant demographics.

Demographic	All	Male	Female	*P‐value*
**Sample size at intake, *n* (%)**				
1995	2401 (51)	859 (36)	1542 (64)	
1997	577 (12)	217 (38)	360 (62)	
1999	1293 (27)	624 (48)	669 (52)	
2001	460 (10)	243 (53)	217 (47)	
Total, *n* (% of cohort)	4731	1943 (41)	2788 (59)	
**Age at intake, mean (SD) (years)**				
1995	81.8 (4.8)	81.6 (4.7)	82.1 (5.0)	
1997	75.0 (1.4)	74.9 (1.2)	75.0 (1.2)	
1999	73.6 (2.4)	73.7 (2.5)	73.4 (2.3)	
2001	71.2 (0.7)	71.2 (0.7)	71.2(0.7)	
Mean (SD)	75.4 (2.3)	77.02 (5.4)	78.3 (5.6)	
**Educational level, *n* (%)**				
No vocational education and −7 years education	385 (9)	124 (32)	261 (68)	<0.001
No vocational education and 7+ years education	1895 (46)	605 (32)	1290 (68)	<0.001
Vocational education and 7–8 years education	845 (21)	551(65)	294 (35)	<0.001
Vocational education and 9+ years education	303 (8)	137 (45)	166 (55)	<0.001
Short and medium education	497 (12)	175 (35)	322 (65)	<0.001
Long education	143 (4)	105 (74)	38 (26)	<0.001
Total, *n* (% of cohort)	4068	1697 (42)	2371(58)	
**Socioeconomic status, *n* (%)**				
Group I (University graduate)	161 (4)	123 (76)	38 (24)	<0.001
Group II (self‐employed 6–20)	448 (11)	247 (55)	201 (45)	<0.001
Group III (self‐employed <5)	814 (20)	570 (70)	244 (30)	<0.001
Group IV (lower level = skill)	805 (20)	410 (51)	395 (49)	<0.001
Group V (unskilled manual)	1200 (30)	347(29)	853 (71)	<0.001
Housewife	640 (15)	0 (0)	640 (100)	<0.001
Total, *n* (% of cohort)	4068	1697 (42)	2371(58)	

Sample size (*n*, %), age (years), educational level and socioeconomic status reported at intake for the entire cohort (all) and for both males and females separately. *P*‐values indicate differences between males and females.

### 3.2 BP and NP

Individual counts of adults reporting no pain, BP, NP, or combined BP and NP for males and females are reported in Table 2. A total of 1196 (25.3%) participants reported BP, 1002 (21.2%) reported NP, and 499 (10.6%) reported combined BP and NP. Females reported a significantly higher prevalence of BP (29.1%), NP (23.1%), and combined BP and NP (12.7%), when compared to males (19.8%, 18.3% and 7.5%, respectively, all *P* ≤ 0.001).

**TABLE 2:** Pain demographics.

Group	All (*n* = 4698)	Male (*n* = 1932)	Female (*n* = 2766)	*P‐value*
No pain, *n* (%)	2500 (25.3)	1190 (19.8)	1310 (29.1)	<0.001
Neck pain, *n* (%)	1002 (21.2)	356 (18.3)	646 (23.2)	<0.001
Back pain, *n* (%)	1196 (25.3)	386 (19.9)	810 (29.1)	<0.001
Combined back and neck pain, *n* (%)	499 (10.6)	146 (7.5)	353 (12.7)	<0.001

Distribution of participants presenting with no pain, back pain, neck pain, and a combined back pain and neck pain reported in the past month for the entire cohort (all), and for both males and females separately. *P*‐values indicate differences between males and females.

### 3.3 Cognitive outcomes

There were no differences in composite cognition scores between those reporting combined BP and NP when compared to those without combined BP and NP for the whole cohort in the unadjusted analysis (34.10 points [95% CI = 35.5, 36.39] vs. 35.80 points [95% CI = 35.44, 36.15]; *P =* 0.129; Table 3). Equally, no differences were observed in males and females reporting combined BP and NP (males 34.38 points [95% CI = 31.88, 36.88]; females 35.72 points [95% CI = 35.19, 36.26]) compared to those with without combined BP and NP (males 35.72 points [95% CI = 35.19, 36.26]; *P* = 0.180; females 35.85 points [95% CI = 35.39, 36.31]; *P* = 0.327; Table 3). While our initial analysis revealed no association between reporting of combined BP and NP and cognitive function in either males or females (Table 3), adjusting for age, sex, socioeconomic and educational status revealed males with combined BP and NP presented with lower composite cognitive scores compared to males without combined BP and NP (81.26 points [95% CI = 73.80, 88.72] vs. 79.48 points [95% CI = 70.31, 88.66]; *P* = 0.043; Table 3). There were no differences in females reporting combined BP and NP compared to those without (87.92 points [95% CI = 79.44, 96.41] vs. 88.03 points [95% CI = 80.70, 95.37]; *P* = 0.850; Table 3).

**TABLE 3:** Composite cognition scores (CCS).

	CCS	95% CI	*P‐value*
Unadjusted			
Without combined BP and NP^*^			
All (*n* = 3665)	35.80	35.44, 31.15	
Males (*n* = 1597)	35.72	35.19, 36.26	
Females (*n* = 2068)	35.85	35.39, 36.31	
**With combined BP and NP**			
All (*n* = 499)	34.10	33.54, 36.39	0.129
Males (*n* = 146)	34.38	31.88, 36.88	0.180
Females (*n* = 353)	35.72	33.45, 36.96	0.327
Adjusted for interview age, sex, socioeconomic and educational status			
Without combined BP and NP^*^			
All (*n* = 3569)	82.22	77.20, 87.24	
Males (*n* = 1551)	81.26	73.80, 88.72	
Females (*n* = 2018)	88.03	80.70, 95.37	
With combined BP and NP			
All (*n* = 499)	81.63	75.65, 87.60	0.223
Males (*n* = 146)	79.48	70.31, 88.66	**0.043**
Females (*n* = 353)	87.92	79.44, 96.41	0.850

Unadjusted and adjusted (for interview, age, socioeconomic and educational status) CCS for the entire cohort (all) and for both males and females separately in those with and without combined BP and NP.

* Group includes those reporting no pain, BP or NP individually. *P*‐values indicate differences between groups.

## 4 DISCUSSION

This cross‐sectional analysis of the LSADT database has provided novel insight into relationships between spinal pain (BP/NP) and cognitive function in elderly Danish twins. We observed no difference in cognition in both males and females reporting spinal pain compared to those reporting no pain. However, contrary to our original hypothesis, we identified that males reporting spinal pain exhibited lower cognitive scores compared to males without spinal pain when adjusting for age, sex, educational and socioeconomic status. This was not observed in the female cohort even though both spinal pain and cognitive impairment are more common in women globally (Dunn et al., 2013; Pinto et al., 2023; Wong et al., 2017). The findings of this study highlight a potential ‘sex‐specific susceptibility’ to cognitive decline in the elderly presenting with spinal pain and supports the notion that spinal pain should be considered as an additional cardio‐cerebrovascular risk factor, warranting further investigation (Rönnegård et al., 2022).

In contrast to our original expectations, females in this cohort reporting a higher prevalence of BP and NP compared to males were not defined by lower cognitive scores in this analysis. These results could be due, in part to a lower (potential) burden of cardiovascular risk factors, despite the potency of sex‐specific risks factors in women (Young & Cho, 2019). It is acknowledged that females report a much higher prevalence of both spinal pain and dementia across the globe (Levine et al., 2021; Wu et al., 2020), which was not evident in the twin cohort in the current study. To what extent the higher prevalence of spinal pain in females contributes to accelerated cognitive decline warrants further consideration and preferably in longitudinal studies where the course of the condition can be mapped and studied. In support, a recent commentary has suggested that females are at the epicentre of a global multimorbidity crisis and are more susceptible to a variety of chronic diseases particularly in later life, including cardiovascular disease, major depression and Alzheimer's disease (Goldstein et al., 2021). Notwithstanding, current evidence suggests that females also exhibit a higher prevalence of early onset symptoms of dementia than males between the ages of 30 and 65 years (Hendriks et al., 2021), and although females may exhibit greater cognitive reserve, that is, a capacity to maintain normal cognitive function in the presence of brain pathology, they exhibit a faster rate of cognitive decline than men (Levine et al., 2021). Even though the global incidence rate of dementia seems to be declining, particularly over the past 25 years, this rate of decline has been more profound in men than women (Wolters et al., 2020).

Several studies have established relationships between chronic pain, physical inactivity, and impaired cognitive function, further supporting the findings of this study (Buchman et al., 2012; Falck et al., 2018; Hamer & Chida, 2009; Thompson et al., 2020). Approximately 60% of older adults do not engage in sufficient levels of physical activity which is required to improve overall cognition and brain health (Tyndall et al., 2018), as low skeletal muscle mass is linked to cognitive decline and dementia in older adults due in part to possible underlying pathophysiological mechanisms related to systemic inflammation, altered insulin metabolism, disturbed protein metabolism and dysfunctional mitochondrial functions (Oudbier et al., 2022). A clear association exists between reduced physical activity levels and the development of a range of chronic health conditions increasing the risk of developing neurodegenerative diseases including dementia (Booth et al., 2017; Hamer & Chida, 2009). In particular, physical activity modulates central nervous system excitability/inhibition and psychological constructs associated with pain and a sedentary lifestyle is associated with greater excitability and less inhibition resulting in more reported chronic pain (Sluka et al., 2018). Furthermore, current evidence suggests that persistent spinal pain may also be associated with neurodegenerative changes in brain structure and responsible for accelerated ageing of the brain in regions responsible for executive function contributing to cognitive decline (Yu et al., 2021).

With a lifetime prevalence rate estimated at 70% (Ramanathan et al., 2018; Rundell et al., 2017; Williams et al., 2018), spinal pain has been closely linked with several comorbidities impacting quality of life (de Luca et al., 2023; Whitlock et al., 2017). Even though we were unable to explore this directly in this study, this association is likely attributed to the disability linked to spinal pain predisposing patients to a more sedentary lifestyle—a primary risk factor contributing to a rise in chronic ill health in later life (Thompson, 2020; Wang et al., 2021). Spinal pain is also associated with cardiovascular disease and cardiac events supporting prior research which demonstrated that chronic pain, particularly spinal pain, is associated with myocardial infarction, stroke and cardiovascular death independent of known cardiovascular risk factors (Fernandez et al., 2016; Rönnegård et al., 2022). Similarly, an association exists between respiratory illness and spinal pain, where emphysema has been identified as a confounding factor for spinal pain and chronic obstructive pulmonary disease has been associated as a risk factor for the development of persistent low back pain (Chen et al., 2017). When considering psychological factors and spinal pain, mental illness is significantly associated with spinal pain (Beynon et al., 2020). This is not surprising as it has long been recognised that the development of persistent spinal pain is strongly associated with depression, anxiety, catastrophizing and low self‐efficacy (Yang et al., 2023), which are also factors considered intermediate in the pathway between experiencing spinal pain and developing long term disability (Lee et al., 2015). Similar psychological symptoms are also present in other chronic pain conditions such as fibromyalgia and osteoarthritis, suggesting common underlying pathophysiological mechanisms related to central sensitisation (Aoyagi et al., 2019; Harte et al., 2018). Other investigations have attributed the adverse impact of spinal pain on the older population to poor psychosocial health and increased medication use (de Luca et al., 2019; Ferreira & de Luca, 2017; Wáng et al., 2016; Whitlock et al., 2017).

The mechanisms related to the pathogenesis of spinal pain and cognitive decline are poorly understood but likely involve a combination of complex interactions between neurotransmitters, oxidative–nitrosative species and cytokines (Chen et al., 2018; Dinakar & Stillman, 2016; Saravanan et al., 2023). While speculative at this stage, we hypothesise two potential mechanisms that may explain the observed relationship between spinal pain and cognitive decline. First, a systemic/local elevation in OXINOS, either as a cause or consequence of spinal pain, may prove the unifying molecular mechanism underlying vascular endothelial dysfunction and structural damage to the neurovascular unit that collectively precede cognitive decline and neurodegeneration (Bailey et al., 2019). Furthermore, OXINOS has the potential to interfere with immunological processes of the brain and accelerate cognitive decline subsequent to microglia activation (Ding et al., 2020; Heneka et al., 2015). In further support, C‐reactive protein, tumour necrosis factor‐α and interleukin‐6 have been shown to be consistently elevated in patients with spinal pain (Li et al., 2016; Pinto et al., 2023; Teodorczyk‐Injeyan et al., 2018, 2011). Second, the musculoskeletal disability and exacerbation of related pain may further promote physical inactivity resulting in a more sedentary existence. This can further compound OXINOS and accelerate cognitive decline subsequent to cerebral hypoperfusion and corresponding reduction in cerebral substrate (oxygen/glucose) delivery (Bailey et al., 2018; Tari et al., 2019). Both (molecular/behavioural) mechanisms could also be the cause or indeed consequence of increased cardio‐cerebrovascular risk factors (i.e., greater vascular disease burden) typically observed (albeit not measured) in males compared to females (Rose et al., 2023). Clearly, further research is encouraged to explore the mechanistic bases underlying the observed relationships reported in this hypothesis‐generating study.

### 4.1 Limitations

There are several limitations to the present study that warrant consideration. The exposure variables (BP and NP) consisted of two simple questions relating to the 1‐month prevalence, which may have underestimated the nature of this complex clinical condition. There was no additional information regarding the duration (acute or chronic), intensity (severity) and frequency of the reported pain to provide any further clinical significance or context, as participants were only asked if they had experienced BP or NP in the last month prior to the interview. Our inability to have better phenotyped pain at interview may have influenced the outcome of the analysis given their potential modulatory impacts on cognitive impairment (Schuler et al., 2004). Notably, it has been suggested that elderly patients with chronic pain may differ from those with acute pain in terms of pain description, and moreover, cognitive impairment appears to alter the ability to localise acute pain, which may impact clinical outcome (Schuler et al., 2004). However, several previous publications have successfully employed this level of data describing spinal pain in elderly Danish twins exploring the potential associations with a range of health confounders, including education, smoking, physical activity and mental functioning (Hartvigsen et al., 2003, 2004, 2006; Leboeuf‐Yde et al., 2009). Second, we were not in a position to formally assess the potential impact of competing comorbidities (and medications) that may have equally impacted the presentation of spinal pain, given that the database prosecuted was pre‐existing and did not capture this important information. Third, the current analysis was a cross‐sectional design and thus we were unable to draw any causal conclusions between spinal pain and cognition. A longitudinal study design would be preferable, as this methodological design detects meaningful changes in variables, (cognition in this case) over time. Fourth, a shortcoming associated with evaluating an elderly population is the potential for a ‘healthy worker effect’, where weaker individuals may have died and therefore have been excluded from the analysis and any tangible association could be overlooked (Chowdhury et al., 2017).

### 4.2 Conclusions

In the current study, males reporting combined BP and NP exhibited lower composite cognitive scores compared to males without combined BP and NP when adjusting for age, sex, educational and socioeconomic status. Given this was only observed in males, these findings may highlight a ‘sex‐specific susceptibility’ to cognitive decline and support the notion that combined BP and NP may be considered as an additional cardio‐cerebrovascular risk factor, warranting further investigation.

## AUTHOR CONTRIBUTIONS

David C. Byfield, Benjamin S. Stacey and Damian M. Bailey had full access to all data in the study and take responsibility for the integrity of data and the accuracy of data analysis. Concept and design: David C. Byfield and Damian M. Bailey; acquisition, analysis and interpretation: All authors; drafting of the manuscript: All authors; critical revision of the manuscript for important intellectual content: All authors. All authors have read and approved the final version of this manuscript. All persons designated as authors qualify for authorship, and all those who qualify for authorship are listed.

## CONFLICT OF INTEREST

D.M.B. is Editor‐in‐Chief of *Experimental Physiology*, Chair of the Life Sciences Working Group, member of the Human Spaceflight and Exploration Science Advisory Committee to the European Space Agency, member of the Space Exploration Advisory Committee to the UK Space Agency, member of the National Cardiovascular Network for Wales and South East Wales Vascular Network and is affiliated to the companies FloTBI, Inc. and Bexorg, Inc. focused on the technological development of novel biomarkers of cerebral bioenergetic function and structural damage in humans.

## Data Availability

The data employed in this retrospective secondary analysis can be accessed upon reasonable request from the corresponding author.
